# CircoTax: a novel circular representation of differentially abundant microbial ranks

**DOI:** 10.1093/femsle/fnag136

**Published:** 2026-11-02

**Authors:** Lorenzo Casbarra, Leandro Di Gloria, Matteo Ramazzotti

**Affiliations:** Dipartimento di Scienze Biomediche, Sperimentali e Cliniche, Università degli Studi di Firenze, viale G.B. Morgagni 50, 50137, Firenze, Italy; Dipartimento di Scienze Biomediche, Sperimentali e Cliniche, Università degli Studi di Firenze, viale G.B. Morgagni 50, 50137, Firenze, Italy; Dipartimento di Scienze Biomediche, Sperimentali e Cliniche, Università degli Studi di Firenze, viale G.B. Morgagni 50, 50137, Firenze, Italy

**Keywords:** novel plot type, microbial taxonomy, differential abundance analysis, rank abundance, software

## Abstract

One of the most relevant applications of next-generation sequencing-based microbial abundance analysis is the discovery of taxa that appear as differentially represented between ecological niches. In the last years several methods have been proposed to accomplish this task, invariantly reporting the differentially represented taxa at the appropriate taxonomic rank, as well as an adjusted *P*-value and a log2 fold change, representing the confidence on differential call and the amount of variation recorded. This work describes CircoTax, a specialized R ggplot2 plot representing a rank-aware collection of differentially abundant taxa in an intuitive, compact radial form. Specifically, the CircoTax is a circular plot with sectors encoding the taxonomy depth (from phylum to genus) and, departing from the center, a number of radial bars that reach rank-based sectors whose color and transparency are proportional to the log fold change intensity direction and *P*-value. The visualization of results is graphically appealing and allows to accommodate tens of differentially abundant ranks each with an associated amount of up or down variation, so that results are easily intelligible for each differentially represented taxa at a glance. The software is freely available and can be run natively in R or through a dedicated ShinyApp for interactive usage.

## Teaser

The CircoTax plot can intuitively represent differentially represented microbial ranks using a novel graphical stategy.

## Introduction

Microbial abundance analysis through next generation sequencing (NGS) is a widely used and largely accepted approach to investigate communities of microorganisms and their relationships or alterations with different environments (Silverman et al. 2020). Quantitative sequencing-based approaches (e.g. RNA-seq, barcode-seq) offer the possibility of quantifying the reads attributed to specific individual molecules in samples, generating a so-called abundance table over which to apply statistical considerations and analytical strategies. Being the observed counts constrained within the total number of reads available for the experiment, the dataset is inherently of compositional nature (Silverman et al. 2020) and, if left untreated, is difficult to analyze with commonly used statistics (Silverman et al. 2020). In addition, the abnormal inflation of zeros is typical of this discipline (Silverman et al. 2020). On the other hand, recent literature proposes several strategies to directly adjust data for classical analyses or to apply non-normal distributions or rank-based analyses to improve data modeling and perform more appropriate investigations (Calgaro et al. 2020, Cappellato et al. 2022, Yang and Chen 2022).

Besides statistical considerations, the analysis of abundance tables may be approached from several perspectives that can generally be divided into multivariate (e.g. clustering or principal coordinate analysis) based on beta diversity estimates aimed at describing the relationships among samples (Paliy and Shankar 2016), or univariate (e.g. detection of differentially abundant taxa), aimed at detecting specific signals or markers that may at least in part explain the observed differences among samples, if any (Cappellato et al. 2022). Eventually, certain comparisons reveal several significant signals, which necessitate the use of space-consuming plots to present them in a more human-readable format, rather than relying on tables rich in numerical values distributed across multiple rows. This holds true especially when the microbiota is explored, as the analysis often includes repeated comparisons across multiple taxonomic levels (phylum, class, order, family, genus, and species). Taxonomic information at different ranks can be obtained by direct comparison of each representative sequence against a closed or open set of pre-taxonomized references (Camacho et al. 2009) or Bayesian inference using k-mers collections (Wang et al. 2007). Motivated by the need of offering an improved yet intuitive representation of the results, we propose a highly informative radial plot, the “CircoTax” plot that exposes differentially abundant signals from abundance tables at the various taxonomic ranks in a compact format. This plot can be generated using publicly available and user-friendly R functions that allow customization. Optionally, we included dedicated functions to offer the flexibility to automate differential analysis through third-party algorithms recognized for their efficiency in handling compositional microbiota data, namely DESeq2 (Love et al. 2014) or ALDEx2 (Fernandes et al. 2013), two largely accepted yet complementary methods, enhancing the efficiency of data handling and results visualization through the CircoTax plot. Fig. 1 adds a graphical perspective on the workflow we implemented to fuel CircoTax plotting. Although not mandatory for usage, the scheme represents a valid starting point to understand the overall CircoTax capabilities.

**Figure 1:**
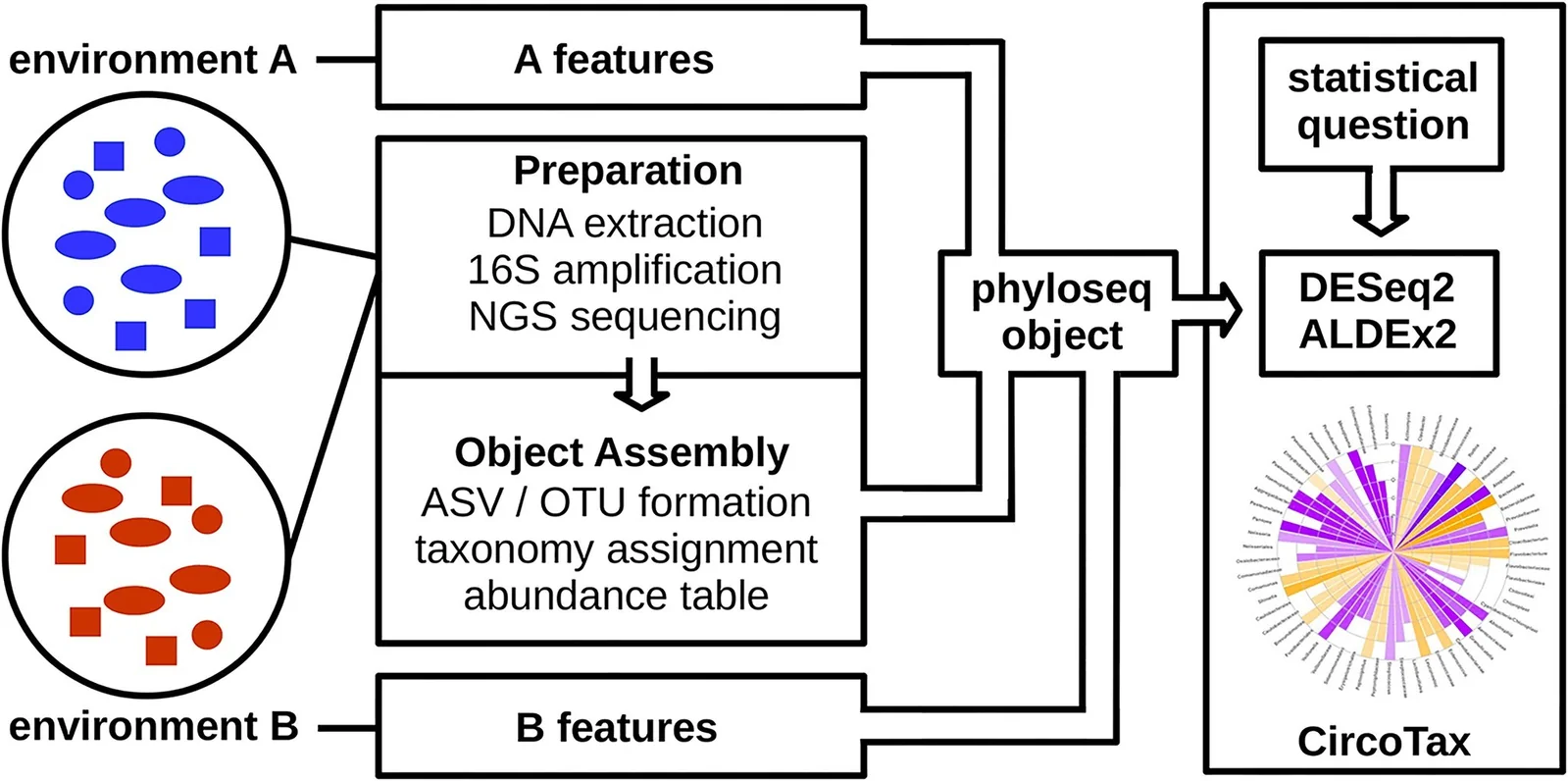
workflow implemented to fuel CircoTax plotting. The R phyloseq object may conveniently store ASV or OTU abundance data that can be “glommed” to specific ranks and statistically tested for differential abundance analysis. CircoTax can receive input from text output of such methods as well as from a properly formatted simple text table (see Table 1 for an example).

## Methods

### CircoTax input data

The CircoTax plotting function basically receives a text table containing in each row a measure of effect size (usually a log fold change) and a (not necessarily complete) taxonomic classification from genus/species to phylum. The effect size (and usually other relevant statistics such as the average signal and the adjusted *P*-value) can be obtained from a plethora of differential analysis methods (Yang and Chen 2022: 130). A final table can be assembled in the statistical language R or loaded from an external file (see the supplementary material for an example). Adding relevant experimental factors associated with samples, a phyloseq (McMurdie and Holmes 2013) R object can be optionally created to host all data in a dedicated framework.

### Taxonomic rank stratification

Our CircoTax function can receive both an input table that combines quantitative differential signals (e.g. abundance fold changes) at various taxonomic ranks (usually phylum, class, order, family, genus and, optionally, species) or a phyloseq object that contains, in phyloseq jargon, otu_table, tax_table, and sample_data (namely the table indicating sample identifiers and their corresponding group). For the scope of CircoTax, the tax_glom function of phyloseq can be used to aggregate counts at a given taxonomic rank by summing the counts of their descendants. This procedure allows us to stratify the differential analyses across every rank. Table 1 summarizes a typical input table of CircoTax. The selection of significant taxa obtained with any user-defined strategy for fold-change and (adjusted) *P*-value filters and their classification depth is different because the analyses are drawn at the different ranks independently. Accordingly, rows 1–2 contain significant results at the phylum level, rows 3–4 at the class level, and so on. An extended version of the table is available as an MS Excel and TXT file (Supplementary Table 1).

**Table 1:** CircoTax input table format. The log2FC column is needed for coloring. The following six columns contain the names of the organisms at the different ranks. The last column is not included in the actual input, rather it has been included to identify the corresponding CircoTax sector. See the supplementary material for a complete version of this table.

log2FC	Domain	Phylum	Class	Order	Family	Genus	Sector
-0.741	Bacteria	Proteobacteria	NA	NA	NA	NA	P
5.294	Bacteria	Chloroflexi	NA	NA	NA	NA	P
-1.221	Bacteria	Proteobacteria	Gammaproteobacteria	NA	NA	NA	C
2.146	Bacteria	Bacteroidetes	Flavobacteriia	NA	NA	NA	C
1.887	Bacteria	Proteobacteria	Alphaproteobacteria	Caulobacterales	NA	NA	O
-3.029	Bacteria	Proteobacteria	Gammaproteobacteria	Pasteurellales	NA	NA	O
2.234	Bacteria	Proteobacteria	Alphaproteobacteria	Caulobacterales	Caulobacteraceae	NA	F
-3.173	Bacteria	Proteobacteria	Gammaproteobacteria	Pasteurellales	Pasteurellaceae	NA	F
4.595	Bacteria	Proteobacteria	Alphaproteobacteria	Rhizobiales	Rhizobiaceae	Shinella	G
2.370	Bacteria	Proteobacteria	Alphaproteobacteria	Caulobacterales	Caulobacteraceae	Brevundimonas	G

### User’s custom input

CircoTax is designed to automate the plotting of differentially abundant taxa at the different ranks starting from a phyloseq object. Nevertheless, CircoTax can also be plugged with a custom data frame according to the user’s own preferences. In that case, the user only needs to specify three columns in a custom data frame (see Table 1 for an example), that is, those referring to taxon names, taxonomic ranks, and the fold change values. There are no particular requirements in this approach: the characters to plot will not be adjusted according to the classical CircoTax syntax and both the names and ranks order simply depend on the order of rows in the data frame.

### Integration of differential analysis tools

The CircoTax plot function comes in a bundle with functions that facilitate differential abundance analysis (DAA). In fact, this information can be achieved in many ways and a large body of literature is available (Calgaro et al. 2020, Yang and Chen 2022, Cappellato et al. 2022). We chose to implement two well-known, relevant, and technically distinct strategies currently used in the microbiota field, namely DESeq2 (Love et al. 2014) and ALDEx2 (Fernandes et al. 2013), both operating in the R framework. The algorithm DESeq2 is designed for RNA-seq counts (Love et al. 2014) but can also be performed on DNA-seq inferred abundances with acceptable robustness and efficiency (Calgaro et al. 2020). In particular, DESeq2 features high sensitivity (Yang and Chen 2022), which allows it to find differences between two similar environments despite maintaining a relatively high false discovery rate, probably due to the different bio-statistical assumptions on the variability of RNA and DNA observations (Lin and Peddada 2020, Russo et al. 2023). On the other hand, the algorithm ALDEx2 features a particularly low false discovery rate and low sensitivity when used on microbiota abundances (Calgaro et al. 2020, Lin and Peddada 2020, Yang and Chen 2022). Providing functions for both the algorithms, the user has the opportunity to choose the best approach for the data under investigation. Our procedure takes advantage of the R phyloseq package (McMurdie and Holmes 2013) to construct (tax_glom) rank-based abundance tables and store sample features (sample_data). We harmonized the input of the two DAA packages so that users can interrogate them with a common, easy to use, and clearly intelligible syntax that respects R formula semantics and accommodates covariates (e.g. for paired analyses). The objective of this effort was to standardize as much as possible (although leaving room for personalization) the retrieval of statistically confident taxonomic ranks to plot. Further details are exposed in the associated manual and example script in the Github repository (see below), which offer practical guidance and step-by-step instructions for applying the described methods.

### Plot design implementation

CircoTax is implemented as an R function that exploits the radial plotting capabilities of the ggplot2 R package for the representation of DAA on microbiota data. The plot is circular, composed by a number of radial sectors that accommodate the information proposed in the table. The taxonomic classification is used to decide how to sort the bars. Several modes are proposed, the main ones being (i) *alphatax*, with members of the same taxonomic lineage that stay close to each other; (ii) *rank*, with bars sorted by rank; and (iii) (abs)*fc*, with the order determined by the value of the log fold change (optionally in absolute value). All sorting options are presented in Fig. 2. Bars receive a color that follows a configurable three-point color scale (defaulting to the colorblind friendly orange-to-white-to-blue scale) so that the opposite changing directions and their intensities can be clearly visualized. The final graph further accommodates a color-scale bar and a title.

**Figure 2:**
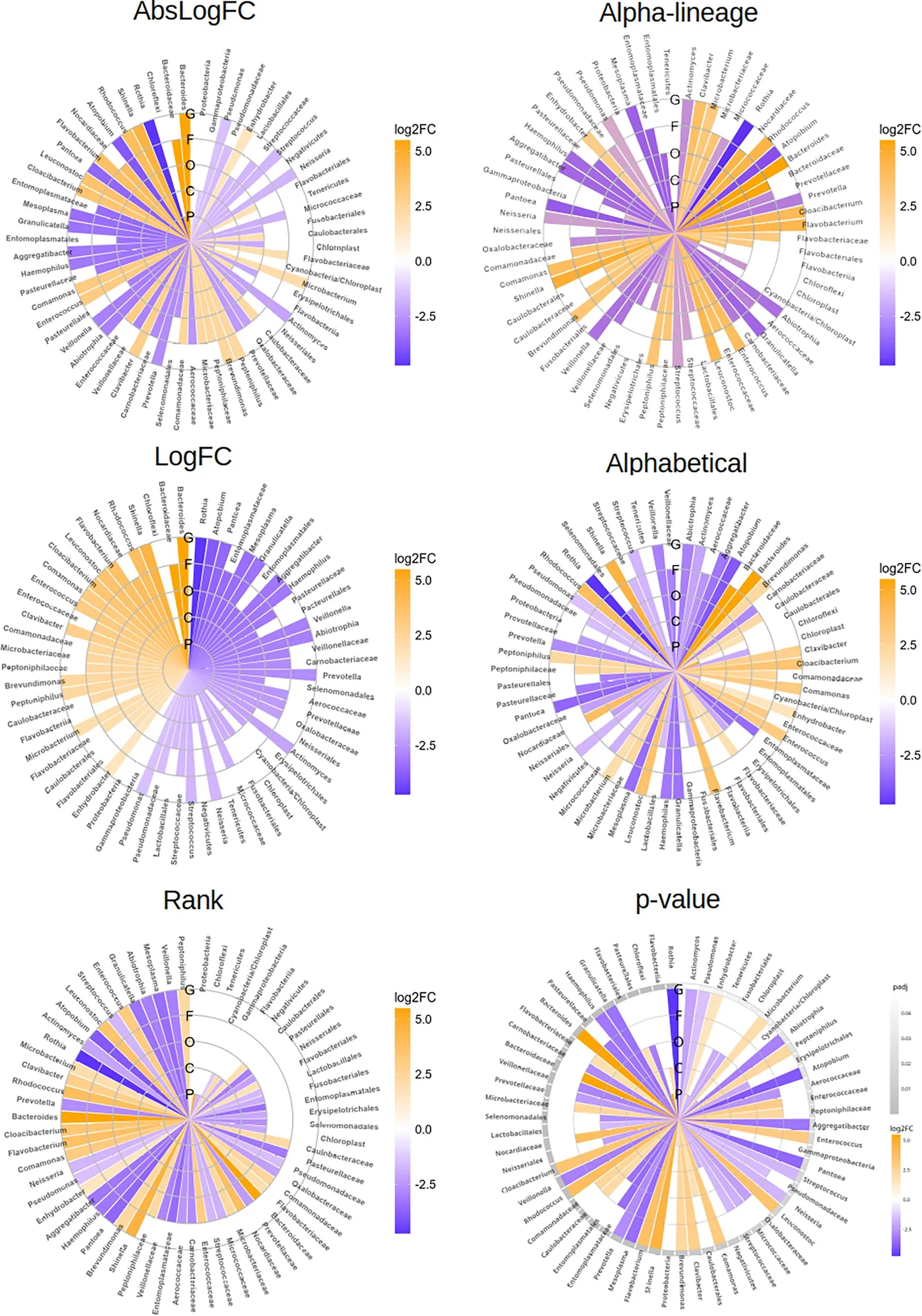
Plotting modes of CircoTax. Plots have a clockwise sorting of bars (sectors) whose ordering can be chosen among “absLogFC” (absolute fold change i.e. with decreasing transparency); “alpha-lineage” (alphabetical order of the highest rank); “LogFC” (log2 fold change); “Alphabetical” (alphabetical order); “Rank” (from genus to phylum) and (adjusted) P-value. The plots refer to a reanalysis of data from Niccolai et al. (Niccolai et al. 2023) and are exposed in tabular format in Supplementary Table 1.

### Testing and validation

CircoTax plots have been already proposed and validated in several datasets previously published by our group (Niccolai et al. 2023, Russo et al. 2023, Baldi et al. 2024, Niccolai et al. 2024). We further included in the GitHub repository (see below), a fully working script that reanalyzes the data from Niccolai et al. (2023) where we documented, for the first time, the presence of a sexually dimorphic breast-associated microbiota suggesting the presence of a cancer-associated dysbiosis in male patients, represented with a CircoTax plot. The choice we made of maintaining full compatibility with the most used packages in the respective fields ensures that CircoTax will provide valid results over time. Furthermore, a convenient ShinyApp has been developed to interact directly with the CircoTax plot, improving its usability. Fig. 3 proposes a comparison between a classic, yet enriched barplot versus the CircoTax plot to properly illustrate the benefits of the radial approach.

**Figure 3:**
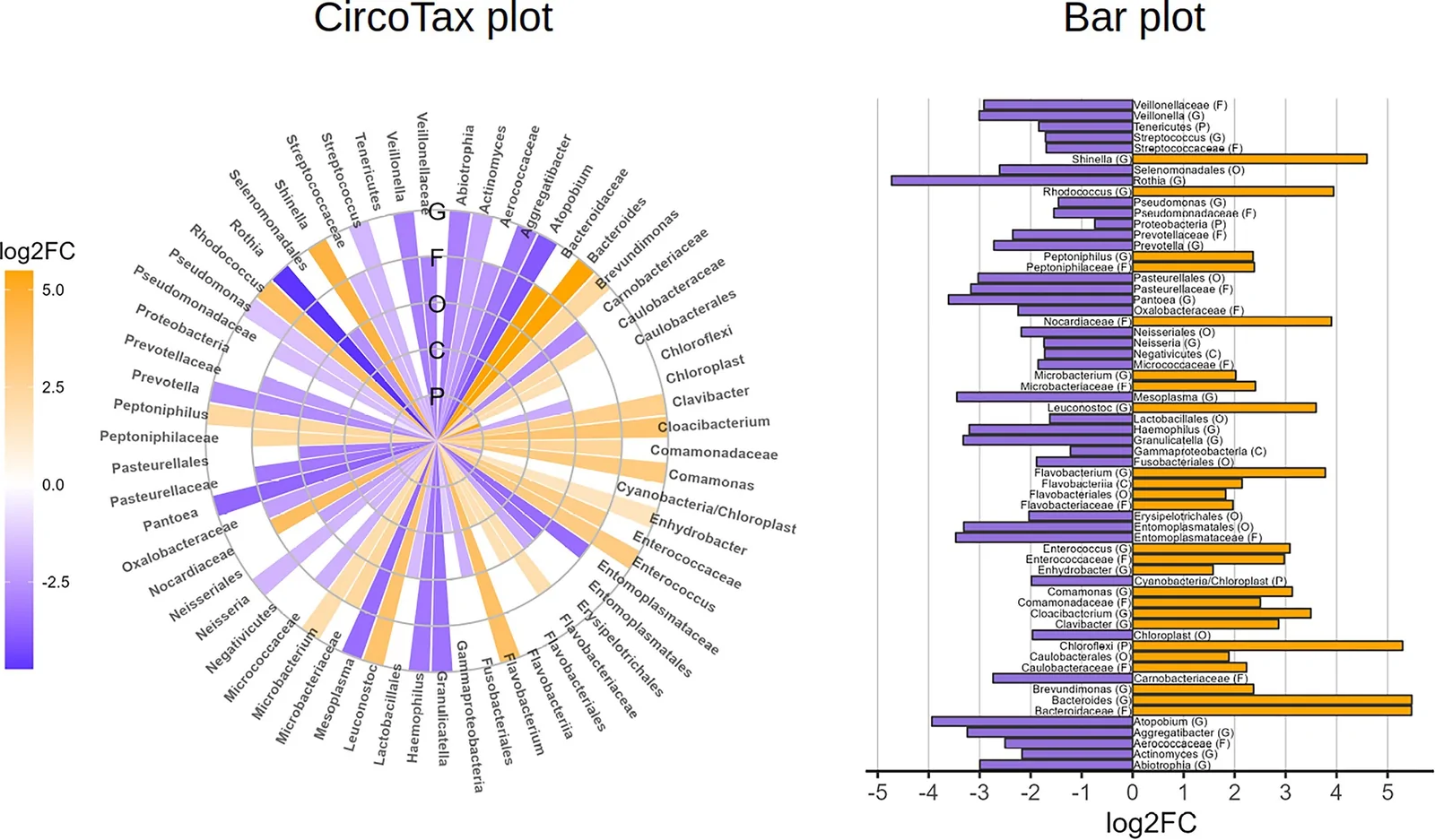
comparison of multi-rank representation using a vertical barplot versus a CircoTax plot.

## Results

### A novel information-rich plot for microbe’s differential abundance analysis

The overall result of CircoTax is a radial barplot in which bars represent the differentially abundant taxa that are stratified in six or seven levels (from domain to genus or species) corresponding to the taxonomic depth and whose color and transparency depend on the log fold change of the differential analysis. This way of representing such results is innovative and intuitive and allows to easily recapitulate the relevant taxonomic ranks discriminating two conditions in a compact form. To exemplify CircoTax capability, Fig. 2 proposes a collection of plot variants that highlights the different sorting options. Such variants are not only relevant to offer alternative perspectives on data but can also disclose hidden peculiarities of the differential analysis results. In fact, each radial bar (sector) (i) reaches a sector that corresponds to a specific rank, making the reader immediately aware of its nature; (ii) has a color and a transparency that clearly depicts the differential trend (more or less abundant) and intensity (how much more or less abundant); and (iii) has a radially oriented label depicting the exact name of the represented element of the rank.

## Discussion

The graphical representation of a differential analysis has been addressed by many strategies (and corresponding packages/programs) before. Most of them use colored bar plots possibly sorted by the (absolute) value of change (e.g. LEfSe; Segata et al. 2011). In this case many panels are needed to represent an entire full rank differential analysis, so figures appearing as part of the results are frequently confined to supplementary data.

Another frequently used representation is based on multiple paired box plots that represent, for each differentially abundant taxonomic rank, relevant metrics (frequently median and IQRs) of the distributions of the two groups under analysis. This is highly informative but, depending on the number of results to be displayed, may require faceting of the figure, making the global picture and the overall message less intuitive.

Sometimes the differential data are used to simply list the interesting taxa and extract the corresponding abundances from an abundance table (e.g. to elaborate a heatmap), possibly reordering the row/columns using distance-based dendrograms. In this case the amount of variation (so the relevance of the change) is lost and the original information is transformed into a complex image with a completely different scope. Anyway, also in this case many panels must be created because the results for different ranks must be elaborated separately. Lastly, since 2011, KronaTools has proposed the Krona graph (Ondov et al. 2011), which is frequently used in web pages to explore interactively the relevant ranks within a dataset through a radial plot. This tool is also used in a static way in recently published publications (Amandito et al. 2022), but in that context its power cannot be fully appreciated due to the staticity of the published paper.

The basic idea and motivation behind the CircoTax plot was to stick with the results of the differential analysis in a way that can cope with both taxonomic relationships among microbes while maintaining a direct view on the amount of change. Interestingly, the radial nature of the plot allows it to accommodate many results at different taxonomic ranks in the same plot that comprehensively represent the full analysis. For this reason, we are convinced that a CircoTax plot may drive information better than current existing strategies.

### Future development

Currently, CircoTax is released as a free-to-use R script that we routinely use in manuscript production together with ancillary scripts that prepare its input tables. Furthermore, a detailed tutorial regarding installation, applications, and technical details is provided. This will be constantly updated to follow the evolution of CircoTax (see the Data availability section). In this release, scripts for the DAA and the graphical representations of its results will be further refined according to user suggestions and novel prespectives. To better cope with different user experiences, the entire procedure has also been prepared and released in the form of an R Shiny website (see below) where phyloseq objects can be received and processed in a coherent environment aimed at deciphering and interpreting the information contained in the datasets.

## Supplementary Material

fnag136_Supplemental_File

## Data Availability

This work reanalyzes data from “Breast cancer: the first comparative evaluation of oncobiome composition between males and females” that are already publicly available in the NCBI Gene Expression Omnibus (GEO) repository at https://www.ncbi.nlm.nih.gov/geo/query/acc.cgi?acc=GSE212891.
